# A 3D inversion method of TEM combining PSO-NLCG optimization and adaptive regularization

**DOI:** 10.1038/s41598-026-48117-x

**Published:** 2026-04-09

**Authors:** Chen Jianqiang, Zhang Feng, Ding Xuhai, Li Yong’en, Zheng Yanhai, Hu Yunfei, Zhang Jun, Yuan Yongbang, Yan Gongbin, He Xin

**Affiliations:** 1 National Energy Group Guoyuan Power Co., Ltd., Beijing, 100033 China; 2 State Key Laboratory of Coal Mine Disaster Prevention and Control, Chongqing, 400037 China; 3 Chongqing Research Institute of China Coal Technology and Engineering Group, Chongqing, 400039 China

**Keywords:** Transient electromagnetic prospecting, 3D visualization inversion, Adaptive regularization, PSO-NLCG hybrid optimization, Engineering, Mathematics and computing, Solid Earth sciences

## Abstract

To enhance the 3D inversion accuracy of transient electromagnetic (TEM) prospecting, a TEM 3D visualization inversion method combining Particle Warm Optimization-Nonlinear Conjugate Gradient(PSO-NLCG) hybrid optimization method and adaptive regularization method is proposed. Based on establish the regularized inversion objective function with the L2 norm, an adaptive parameter adjustment strategy for the regularization objective function is proposed to optimize the regularization parameters and improve the stability of the inversion. To further ensure the stability of the inversion computation, a dynamic adjustment mechanism between data fitting and model smoothing is constructed, and the upper and lower bounds constraints and inversion termination conditions are formulated. To improve computation speed and inversion accuracy of 3D TEM inversion, based on the theory of gradient optimization algorithms, the global search capability of the particle swarm optimization (PSO) algorithm is combined with the refined exploration of the nonlinear conjugate gradient (NLCG) algorithm to form the PSO-NLCG hybrid optimization algorithm. The results of the comparative experiments show that, the proposed PSO-NLCG hybrid optimization and adaptive regularization algorithm for TEM 3D inversion can effectively recover the spatial distribution of underground anomalous target bodies, and the inversion accuracy is higher than that of the comparative method.

**Supplementary Information:**

The online version contains supplementary material available at 10.1038/s41598-026-48117-x.

## Introduction

The transient electromagnetic method (TEM) has demonstrated significant potential in various applications, including shallow surface engineering geological surveys, groundwater structures, detection urban underground space, and metallic resource prospecting, due to the increasing demand for geophysical exploration^1–4^. TEM measurements are often affected by strong background noise, power-line interference, and instrumental noise during data acquisition. These interference effects lead to a reduced Signal-to-Noise Ratio (SNR) and decreased data accuracy, which directly degrade the reliability and resolution of 3D TEM inversion^5,6^. Although traditional filtering methods^7,8^ and deep learning techniques^9–11^ can improve signal quality to some extent, achieving a good balance between denoising performance and feature preservation remains challenging. Furthermore, 3D TEM inversion is inherently nonlinear and ill-posed, involving high computational complexity. Consequently, its accuracy, stability, and efficiency remain limited. Considerable efforts have been devoted to developing more effective inversion frameworks. In particular, intelligent optimization algorithms have been employed to enhance inversion performance, while advanced visualization techniques have been introduced to improve the interpretability of the results.

In terms of theoretical modeling for 3D TEM inversion, Liu et al.^12^ proposed a unified inversion framework applicable to various loop-source configurations for 3D TEM modeling and inversion. The approach exhibited strong adaptability and stability by constructing a multi-source excitation response model capable of handling complex geological conditions. This work provided theoretical and practical foundations for the inversion and analysis of multi-source TEM data. Wang et al.^13^ developed a 3D forward modeling and inversion scheme for transient electromagnetic data considering inhomogeneous magnetic permeability. The method, based on a finite-element formulation and implicit time-stepping, achieved higher accuracy in complex magnetic environments. Consequently, this work established a robust foundation for performing inversion in realistic geological settings.

Regarding objective function design, Sun et al.^14^ incorporated optimal transport theory and formulated an inversion objective function based on the Wasserstein distance. The proposed approach exhibits greater sensitivity and robustness when dealing with complex anomaly boundaries compared to the traditional L2 norm. This enhances the capability for detailed local imaging. However, challenges such as limited stability and susceptibility to local minima remain in 3D TEM inversion. Xue et al.^15^ pointed out in a recent review that current global optimization strategies and adaptive regularization parameter schemes in 3D TEM inversion remain inadequate. Therefore, developing more robust and generalizable inversion frameworks is essential to overcome these limitations and enhance the reliability of 3D TEM inversion.

To address the challenges associated with nonlinearity, ill-posedness, and high computational complexity in 3D TEM inversion, Xiao et al.^16^ developed an efficient inversion framework based on Octree adaptive finite element meshes to address the challenges of nonlinearity, ill-posedness, and high computational complexity in 3D TEM inversion. This greatly enhanced modeling accuracy and computational efficiency. Yang et al.^17^ introduced vector finite elements and unstructured meshes to enable accurate semi-airborne data modeling under complex terrain conditions. He et al.^18^ proposed a hybrid inversion strategy that integrates particle swarm optimization with gradient descent. This enhanced global search capability for multi-modal problems while maintaining stable convergence. Lei et al.^19^ proposed a parallel inversion method employing an approximate Jacobian matrix to improve computational efficiency in large-scale applications. Yi et al.^20^ proposed a joint strategy that combines frequency–time domain transformation with multi-source modeling for ground-source airborne-receiver time-domain data. This significantly enhanced the resolution of geological boundaries.

At the level of forward modeling and optimization algorithms, Hajebi et al.^21^ reviewed stochastic optimization techniques for subsurface inverse profiling and imaging, and reported that methods such as CMA-ES and Evolutionary Programming can achieve faster convergence and higher solution quality than PSO in certain geophysical inversion settings. Zhang et al.^22^ proposed a triple-grid framework for primary field simulation, forward response computation, and Jacobian matrix estimation within forward modeling and optimization processes. This strategy significantly reduced memory usage and computational cost. Cai et al.^23^ developed an effective 3D TEM inversion method based on the finite-element approach integrated with a parallel direct solver. This method enhanced computational performance and enabled efficient processing of large-scale airborne electromagnetic datasets, thereby facilitating faster and more accurate subsurface imaging. Su et al.^24^ formulated a 3D anisotropic inversion model to improve the characterization of heterogeneous and structurally complex geological formations. Lu et al.^25^ proposed a pseudo-wavefield imaging approach for TEM data based on a sweep-time-preconditioned precise integration algorithm. The approach enhanced lateral resolution by simulating wave-equation-based propagation of transient electromagnetic fields. Yu et al.^26^ developed a deep learning–based inversion method employing convolutional neural networks for airborne time-domain electromagnetic data. This enabled an efficient, accurate inversion workflow and showed potential for enhancing 3D TEM imaging and interpretation.

Furthermore, to enhance the interpretability and visualization of 3D inversion results, researchers have progressively adopted 3D modeling and visualization platforms. Parquer et al.^27^ validated the consistency of 3D geological models by integrating topological rules with geometric verification, which enhanced model reliability and structural coherence. Puntu^28^ and Wang^29^ realized 3D visualization of subsurface structures by integrating multi-resistivity and TEM data with machine learning, statistical modeling, and kriging-based interpolation techniques. These approaches significantly improved the delineation of geological and redox structure boundaries. With the advancement of virtual reality and graphics rendering technologies, such methods offer enhanced accuracy and interpretability for complex geological environments. Tian^30^ and Domenzain^31^ developed 3D visualization platforms supporting interactive operations and large-scale data processing. These developments transitioned TEM modeling from static display to immersive, multidimensional interaction.

In summary, 3D TEM inversion remains challenged due to strong nonlinearity, ill-posedness, and multimodal objective functions, leading to sensitivity to initial models and regularization parameters. Gradient-based methods such as NLCG exhibit fast local convergence but are prone to local minima, whereas global optimization methods like PSO provide robust global exploration with high computational cost when used alone. To exploit their complementary strengths, this study proposes a hybrid PSO-NLCG framework, where PSO performs global search to obtain reliable initial solutions and NLCG enables efficient local refinement. Furthermore, an adaptive regularization strategy is incorporated to dynamically balance data misfit and model smoothness, thereby enhancing inversion stability, accuracy, and the reconstruction of subsurface anomaly geometry, supported by integrated 3D visualization inversion.

## Method principle

### Regularized $d=F\left( m \right)+e$ TEM inversion

#### Inversion objective function and its gradient

The forward problem of the TEM can be formulated as follows:

1 $$d=F\left( m \right)+e$$

where *d* represents the observed data (i.e., the transient electromagnetic response); *F* denotes the nonlinear forward operator, which is derived by discretizing Maxwell’s equations using the finite element method (FEM); and *e* represents the observational noise.

The TEM observation data vector and the inversion model parameter vector are defined as:

2 $$\left\{ {\begin{array}{*{20}{l}} {d={{\left[ {{d_1},{d_2}, \ldots ,{d_{{N_d}}}} \right]}^T}} \\ {m={{\left[ {{m_1},{m_2}, \ldots ,{m_{{N_m}}}} \right]}^T}} \end{array}} \right.$$

where ${N_d}$ is the dimension of the observation data vector, ${N_m}$ is the dimension of the model parameter vector, and ${m_i}$ represents the conductivity parameter (logarithmic conductivity) at the discretized element (mesh node). The objective of the inversion problem is to determine *m s*uch that the simulated response data obtained by forward modeling closely matches the observed data.

The 3D visualization inversion of TEM data is an ill-posed problem. The observed data usually contain noise, and the inversion results may be unstable or non-unique. Regularization constraints are essential for stabilizing the solution of TEM inversion. The core idea is to introduce constraints such as model smoothness or reference models to mitigate the ill-posedness and suppress the influence of noise. Therefore, the inversion objective function is expressed as a weighted sum of the data misfit term and the regularization term:

3 $${\phi _{(m)}}={\phi _d}(m)+\lambda {\varphi _m}$$

In the equation, ${\phi _d}(m)$ represents the data misfit term, which measures the discrepancy between the response predicted by the model *m* and the observed data; ${\varphi _m}$ denotes the regularization (model) constraint term; and $\lambda$ is the regularization parameter used to balance the weights between data fitting and model smoothness. In this study, an objective function based on the ${L_2}$ norm is adopted. Thus, the data misfit term${\phi _d}(m)$ is written as:

4 $${\phi _d}(m)=\left\| {{W_d}\left[ {{d^{obs}} - F(m)} \right]} \right\|_{2}^{2}={\left[ {{d^{obs}} - F(m)} \right]^T}W_{d}^{T}{W_d}\left[ {{d^{obs}} - F(m)} \right]$$

${W_d}$ denotes the data weighting matrix, where its diagonal elements are defined as ${\left( {Wd} \right)_{ii}}=\frac{1}{{{\sigma _\iota }}}$, with ${\sigma _i}$ representing the standard deviation of the $i$-th observed data. ${d^{obs}}$ is the observed data vector of dimension ${N_d} \times 1$, and $F(m)$ represents the predicted data calculated through forward modeling using the model *m*.

The regularization term for model constraints is defined as:

5 $${\phi _m}=\left\| {{W_m}\left[ {m - {m^{ref}}} \right]} \right\|_{2}^{2}={\left[ {m - {m^{ref}}} \right]^T}W_{m}^{T}{W_m}\left[ {m - {m^{ref}}} \right]$$

where *m* and ${m^{ref}}$ denote the computed model parameter vector and the reference model vector, respectively. ${W_m}$ is the model weighting matrix. In this study, a minimum-smoothness model constraint is adopted. The matrix ${W_m}$ can be obtained by discretizing the Laplacian operator, as shown in Eq.(6).

6 $${W_m}={\alpha _x}{W_x}+{\alpha _y}{W_y}+{\alpha _z}{W_z}$$

where ${\alpha _x}$, ${\alpha _y}$ and ${\alpha _z}$ denote the smoothing coefficients of the discrete Laplacian matrix in the *x*, *y*, and *z* directions, respectively, and ${W_x}$, ${W_y}$, and ${W_z}$ represent the second-order difference matrices in the corresponding directions. The matrix ${W_x}$ is given by:

7 $${W_x}=\left( {\begin{array}{*{20}{c}} {\frac{{\sqrt {{V_1}} }}{{{h_1}{h_1}}}}&{ - \left( {\frac{{\sqrt {{V_1}} }}{{{h_1}{h_1}}}+\frac{{\sqrt {{V_1}} }}{{{h_2}{h_1}}}} \right)}&{\frac{{\sqrt {{V_1}} }}{{{h_2}{h_1}}}}&{...}&{...}&{...} \\ {...}&{...}&{...}&{...}&{...}&{...} \\ {...}&{...}&{\frac{{\sqrt {{V_i}} }}{{{h_i}{h_i}}}}&{}&{ - \left( {\frac{{\sqrt {{V_i}} }}{{{h_i}{h_i}}}+\frac{{\sqrt {{V_i}} }}{{{h_{i+1}}{h_i}}}} \right)}&{\frac{{\sqrt {{V_i}} }}{{{h_{i+1}}{h_i}}}} \end{array}} \right)$$

where ${h_i}$ denotes the distance between the centers of two adjacent elements in the $x{\mathrm{-}} dircetion$, and ${V_i}$ represents the volume of the $i {\mathrm{-}} th$ hexahedral element.

Taking the first-order derivative of the objective function (Eq. (3)) with respect to the model parameters *m* yields the gradient of the objective function:

8 $$g=\frac{{\partial {\phi _d}}}{{\partial m}}+\lambda \frac{{\partial {\varphi _m}}}{{\partial m}}= - 2{J^T}W_{d}^{T}{W_d}r+2\lambda W_{m}^{T}{W_m}\left( {m - {m^{ref}}} \right)$$

where $r={d^{obs}} - F(m)$ and *J* are sensitivity matrices, defined as follows:

9 $$J=\frac{{\partial F(m)}}{{\partial m}}=\left[ {\begin{array}{*{20}{c}} {\frac{{\partial {F_1}}}{{\partial {m_1}}}}& \cdots &{\frac{{\partial {F_1}}}{{\partial {m_{{N_m}}}}}} \\ \vdots & \ddots & \vdots \\ {\frac{{\partial {F_{{N_d}}}}}{{\partial {m_1}}}}& \cdots &{\frac{{\partial {F_{{N_d}}}}}{{\partial {m_{{N_m}}}}}} \end{array}} \right]$$

#### Bound constraints and adaptive regularization strategy

To ensure that the estimated model parameters remain non-negative and within a reasonable range, bound constraints are imposed on the model parameters:

10 $${m_k}=\log ({\rho _K} - a) - \log (b - {\rho _K}), \quad a<{\rho _K}<b$$

where $k=1,2, \ldots ,{N_m}$, with *a* and *b* denoting the prescribed lower and upper bounds of the model parameters. The inversion is carried out by iteratively updating *m*, which is subsequently transformed into resistivity values via Eq. (11) upon completion.

11 $${\rho _K}=\frac{{a+b{e^{{m_k}}}}}{{1+{e^{{m_k}}}}}$$

Regularization constraints are incorporated into the objective function to suppress unstable solutions in the TEM 3D visualization inversion. However, fixed regularization constraints are often insufficient under complex geological conditions, because they fail to accommodate the varying requirements across different inversion stages. To address this issue, an adaptive regularization strategy is adopted, in which the regularization factor is dynamically adjusted according to model updates to enhance inversion stability. At the early stage of inversion, a relatively large regularization factor is applied to enforce stronger constraints, thereby suppressing model complexity and ensuring optimization stability. As the number of iterations increases, the regularization factor is gradually reduced, enabling the inversion algorithm to refine the solution with reduced smoothness constraints and to improve the resolution of the final model. The adjustment of the regularization factor follows an exponential decay function, defined as follows:

12 $$\lambda ={\lambda _0} \times {e^{\left( { - \delta \cdot (k - 1)} \right)}}$$

where ${\lambda _0}$ is the initial regularization factor, typically chosen as a relatively large value; $\delta$ is the decay rate; and $k\left( {k=1,2, \ldots ,M} \right)$ denotes the current iteration number.

#### Termination criteria

The convergence of the inversion process is evaluated using the root-mean-square (RMS) error as the termination criterion, given by:

13 $$RMSE = \sqrt {\frac{1}{N}\sum\limits_{{i = 1}}^{{N_{d} }} {\left( {\frac{{d_{i}^{{obs}} - F_{i} (m)}}{{\sigma _{i} }}} \right)^{2} } }$$

The RMSE is used to evaluate the difference between observed data and the model’s predicted response. When the RMSE drops below a preset threshold, the inversion is deemed to have achieved sufficient data fitting, and the process terminates. However, if the inversion process stagnates in a local minimum, the gradient norm is introduced as an alternative convergence criterion, defined as:

14 $${\left\| g \right\|^2}<\varepsilon$$

$\varepsilon$ is a sufficiently small positive constant. When the gradient value falls below this threshold, it indicates that changes in the inversion model have become negligible. Continuing the iteration would lead to unnecessary computational cost, and the inversion process should be terminated. The specific value of $\varepsilon$ can be adjusted based on the characteristics of the problem and the chosen inversion algorithm.

### The PSO-NLCG hybrid optimization algorithm

#### The PSO algorithm

The PSO primarily simulates the cooperative behavior among individuals within a biological population, employing multiple particles to perform a parallel search in the solution space, thereby preventing premature convergence to local optima. In the global search phase, the population initialization is defined as:

15 $${X_{i,j}}^{0}=l{b_j}+R \times \left( {u{b_j} - l{b_j}} \right),\quad i=1,2, \ldots ,N,\quad j=1,2, \ldots ,dim$$

where ${X_{i,j}}^{0}$ represents the value of the $j {\mathrm{-}} th$ variable of the $i {\mathrm{-}} th$ particle. *N* denotes the population size, $dim$ is the dimensionality of the problem, *R* is a random number uniformly distributed in the range [0, 1], and $l{b_j}$ and $u{b_j}$ represent the lower and upper bounds, respectively.

After the initialization process, the fitness value $\phi \left( {{X_{i,j}}^{0}} \right)$ of each particle is calculated based on the objective function, and both the individual best and the global best positions are updated accordingly. Each particle updates its velocity and position according to the following equation:

16 $$\left\{ {\begin{array}{*{20}{l}} {V_{i}^{{k+1}}=\omega \cdot V_{i}^{k}+{C_1} \times {r_1} \times \left( {pbest_{i}^{k} - X_{i}^{k}} \right)+{C_2} \times {r_2} \times \left( {gbest_{i}^{k} - X_{i}^{k}} \right)} \\ {X_{i}^{{k+1}}=X_{i}^{k}+V_{i}^{{k+1}}} \end{array}} \right.$$

where $\omega$ represents the inertia weight that controls the exploration ability of the particles (set to 0.8 in this study). ${C_1}$ and ${C_2}$ are the cognitive and social learning factors, typically set to ${C_1}={C_2}=2$; $i=1,2,3, \ldots ,N$ denotes the iteration index. *N* is the total number of particles. ${r_1}$ and ${r_2}$ are random numbers uniformly distributed in the interval (0, 1). $pbest_{i}^{k}$ and $gbest_{i}^{k}$ denote the personal best position of the $i {\mathrm{-}} th$ particle and the global best position among all particles, respectively. $X_{i}^{k}$ is the current position of the particle (the current model parameters). $V_{i}^{{k+1}}$ is the particle velocity (the update amount of the model parameters), with a maximum value of ${V_{max}}>0$. if $V_{i}^{k}>{V_{max}}$, then $V_{i}^{k}$ is set to ${V_{max}}$. After completing I iterations, the PSO algorithm outputs the globally optimal solution obtained through the search process.

### The nonlinear conjugate gradient method

The nonlinear conjugate gradient (NLCG) method is an extension of the conventional conjugate gradient algorithm, designed to solve general nonlinear optimization problems. In each iteration, since the objective function is non-quadratic, the gradient must be recalculated, and a line search is performed to determine the step size.

Step 1: Initialization

Set the initial inversion model ${m_0}$, and compute the initial gradient:

17 $${g_0}=\nabla \phi ({m_0})$$

Set the initial search direction to the negative gradient, ensuring that the initial search proceeds along the direction of steepest descent. The specific formula is given as:

18 $${d_0}= - {g_0}$$

For the $k {\mathrm{-}} th$ iteration, given the current model ${m_k}$, gradient ${g_k}$, and search direction ${d_k}$, perform the following updates:

Step 2: Step size calculation:

Similar to the classical Conjugate Gradient method, the NLCG also requires computation of a step size ${\alpha _k}$. However, due to the nonlinearity of the objective function, ${\alpha _k}$ is determined via a line search. Specifically, the algorithm searches along the direction ${d_k}$ for a step size ${\alpha _k}$ that minimizes the objective function:

19 $$\alpha _{\kappa } = \arg \mathop {\min }\limits_{\alpha } \phi \left( {m_{k} + \alpha d_{k} } \right)$$

Additionally, the step size must satisfy the Wolfe conditions:

20 $$\phi \left( {{m_k}+{\alpha _k}{d_k}} \right) \leqslant \phi \left( {{m_k}} \right)+{c_1}{\alpha _k}\nabla \phi {\left( {{m_k}} \right)^T}{d_k}|\nabla \phi {\left( {{m_k}+{\alpha _k}{d_k}} \right)^T}{d_k}| \leqslant {c_2}|\nabla \phi {\left( {{m_k}} \right)^T}{d_k}$$

where ${c_1}$ and ${c_2}$ are predefined constants, typically satisfying $0<{c_1}<{c_2}<1$.

Step 3: Solution update and gradient recalculation:

Update the model parameters using the obtained step size ${\alpha _k}$:

21 $${m_{k+1}}={m_k}+{\alpha _k}{d_k}$$

Compute the new gradient at the updated model:

22 $${g_{k+1}}=\nabla \phi ({m_{k+1}})$$

Step 4: Conjugate coefficient calculation and search direction update:

A key step in the Nonlinear Conjugate Gradient method is the computation of the conjugate coefficient ${\beta _k}$, which ensures that the new search direction incorporates both the current gradient information and the influence of the previous search direction. Common formulas for ${\beta _k}$ include:

Fletcher–Reeves (FR) formula:

23 $${\beta _k}^{{FR}}=\frac{{{{\left\| {{g_{k+1}}} \right\|}^2}}}{{{{\left\| {{g_k}} \right\|}^2}}}$$

Polak–Ribière (PR) formula:

24 $${\beta _k}^{{PR}}=\frac{{{g_{k+1}}^{T}\left( {{g_{k+1}} - {g_k}} \right)}}{{{{\left\| {{g_k}} \right\|}^2}}}$$

Based on the computed conjugate coefficient, the update formula is a as follows:

25 $${d_{k+1}}= - {g_{k+1}}+{\beta _{k+1}}{d_k}$$

In some cases, if the search direction loses conjugacy, it may be reset to the negative gradient direction, i.e., ${d_{k+1}}= - {g_{k+1}}$.

The above steps are repeated until $\left\| {{g_{k+1}}} \right\|<\varepsilon$ becomes sufficiently small or the maximum number of iterations is reached. The current model ${m_{k+1}}$ is then returned as the final solution.

#### The procedure of the PSO-NLCG hybrid optimization algorithm

**Fig. 1:**
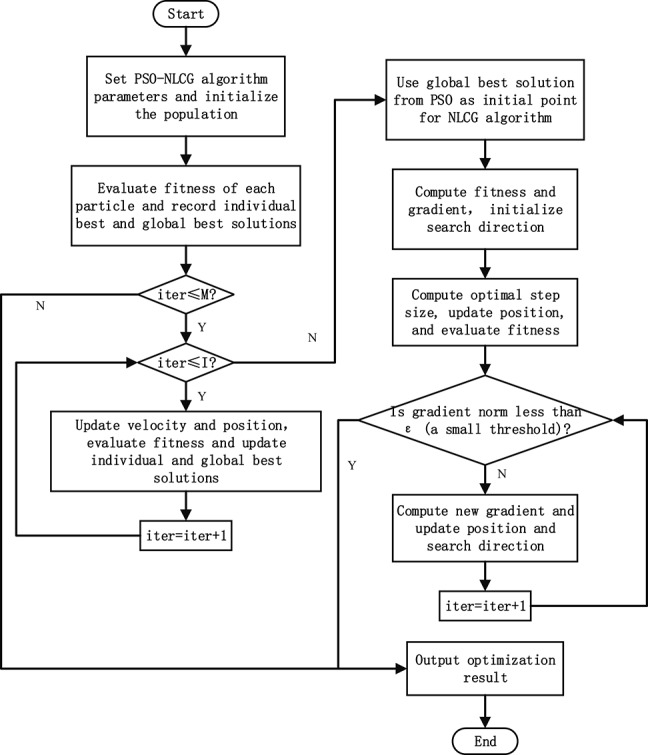
The procedure of the PSO-NLCG hybrid optimization algorithm.

The PSO-NLCG framework employs a two-stage optimization strategy. The first stage corresponds to the global search phase, where the swarm intelligence-based PSO algorithm is employed during the initial iterations to explore the broader solution space and identify promising global regions. The second stage corresponds to the local refinement phase, in which the global solution obtained from the PSO stage is used as the initial point, and the NLCG algorithm is applied for local optimization to achieve fine-tuning of the solution. The procedure of the PSO–NLCG hybrid optimization algorithm is shown in Figure 1. The detailed implementation procedure is summarized as follows:

Step 1: Parameter initialization

The problem dimensionality $dim$, the upper and lower bounds of the search space, the maximum number of iterations *M*, the population size *N*, the maximum number of iterations for the PSO stage *I*, and predefined convergence thresholds such as $\varepsilon$ are first defined. Algorithm parameters, including the inertia weight $\omega$, the cognitive learning factor ${C_1}$, and social learning factor ${C_2}$, are then determined.

Step 2: Population initialization

The initial particle population is generated according to Eq. (15). The fitness value of each particle is then evaluated based on the inversion objective function, after which the particles are ranked accordingly. Both the individual best solution $pbest$ and the global best solution $gbest$ are subsequently recorded.

Step 3: Global search

Within the predefined number of iterations *I*, the PSO algorithm is employed to perform the global search. By continuously updating the particle positions and velocities, the swarm is directed toward the optimal region of the solution space. Particles that exceed the boundaries are reset, and their fitness values are subsequently re-evaluated. The individual and global best solutions are then updated accordingly to ensure continuous improvement of the parameter estimates.

Step 4: NLCG initialization

The global best solution obtained from the $I {\mathrm{-}} th$ iteration of the PSO stage is set as the initial point for the local search. At this point, the fitness value is evaluated, and the gradient is computed. The initial search direction is then defined as the negative gradient vector.

Step 5: Local exploration

The step size is determined using Eq. (20), followed by an update of the model parameters. The new fitness value and gradient are then computed using Eqs. (21) and (22), respectively, and the current best fitness value is recorded. Subsequently, the conjugate coefficient ${\beta _k}$ is calculated using Eq. (23), and the search direction is updated according to Eq. (25).

Step 6: Convergence check and termination criteria

The norm of the gradient is examined to determine whether it falls below the predefined threshold $\varepsilon$. If this condition is satisfied, the local optimization is regarded as converged, and the iteration process is terminated. In addition, the algorithm checks whether the maximum number of iterations *M* has been reached. If so, the optimization process is terminated; otherwise, the procedure returns to Step 5 to continue the local search (Fig. 1).

### Performance evaluation of the PSO-NLCG hybrid optimization algorithm

**Fig. 2:**
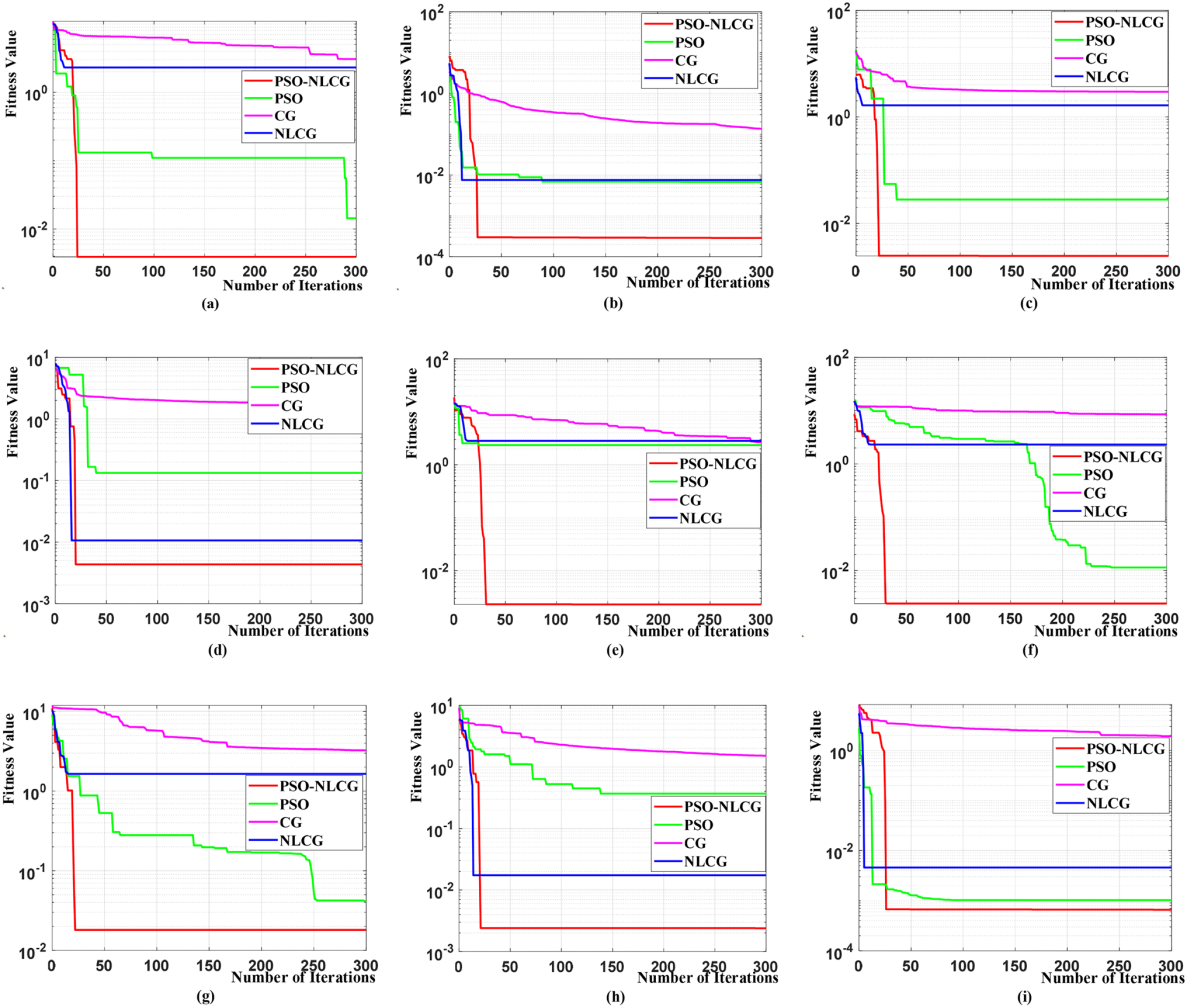
Convergence curves of different algorithms on benchmark functions. Subfigures (**a**)–(**i**) correspond to the convergence plots for benchmark functions F1 to F9, respectively.

**Fig. 3:**
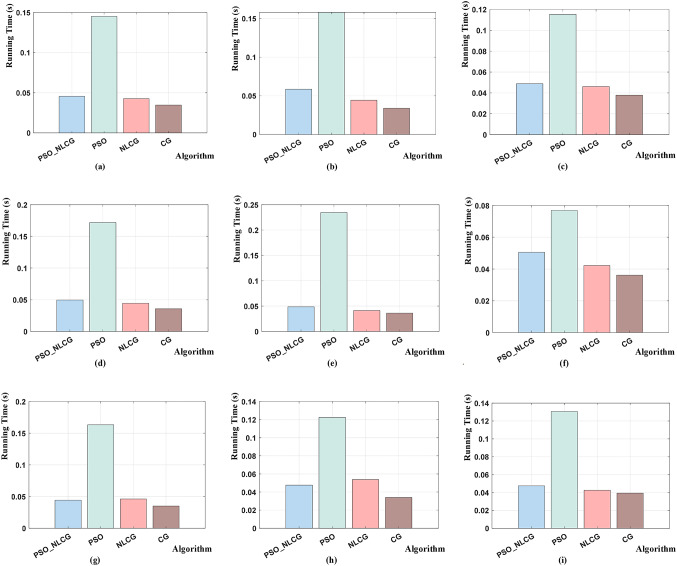
Comparison of running time for different algorithms on benchmark functions. Subfigures (**a**)–(**i**) correspond to the running times for benchmark functions F1 to F9.

To evaluate the performance of the proposed hybrid optimization algorithm, nine benchmark functions were selected for comparative testing among the PSO-NLCG, NLCG, CG, and PSO algorithms. Table 1 summarizes the benchmark functions and their search-space bounds (upper and lower limits), which are consistently used as the variable constraints for all compared algorithms. To minimize the influence of randomness on the results, each algorithm was executed eleven times, and the median performance was adopted. For a fair comparison, the population size was set to 100 for the population-based methods (PSO-NLCG and PSO), and the maximum number of iterations was set to 300 for all methods (PSO-NLCG, NLCG, CG, and PSO). The final optimization results and execution times of all algorithms are summarized in Tables 2 and 3, respectively. In addition, the convergence curves and runtime comparisons are illustrated in Figs. 2 and 3.

**Table 1:** Search space bounds on benchmark functions.

No.	Function Name	Range	Theoretical optimum	Function type
F1	Rosenbrock	[− 32,32]	0	Unimodal
F2	Quartic	[− 1.28,1.28]	0	Unimodal
F3	Sphere	[− 100,100]	0	Unimodal
F4	Rastrigin	[− 5.12,5.12]	0	Multimodal
F5	Griewank	[− 600,600]	0	Multimodal
F6	Ackley	[− 32, 32]	0	Multimodal
F7	Alpine	[− 10,10]	0	Multimodal
F8	Schwefel	[− 10,10]	0	Multimodal
F9	Kowalik’s	[− 5,5]	0	Multimodal

**Table 2:** Optimization results comparison on benchmark functions.

No.	Function name	Function type	Theoretical optimum	PSO-NLCG	NLCG	CG	PSO
F1	Rosenbrock	Unimodal	0	3.9e−3	2.3168	3.0654	1.43e−2
F2	Quartic	Unimodal	0	2.8713e−4	7.6e−3	1.353e−1	6.8e−3
F3	Sphere	Unimodal	0	2.5e−3	1.6462	2.9460	2.828e−2
F4	Rastrigin	Multimodal	0	4.3e−3	1.06e-2	1.7460	1.326e−1
F5	Griewank	Multimodal	0	2.3e−3	2.8143	2.6614	2.3582
F6	Ackley	Multimodal	0	2.4e−3	2.3168	8.5228	1.14e−2
F7	Alpine	Multimodal	0	1.80e−2	1.6462	3.2425	4.07e−2
F8	Schwefel	Multimodal	0	2.4e−3	1.74e−2	1.5165	3.670e−1
F9	Kowalik’s	Multimodal	3E-4	6.5991e−4	4.6e−3	1.9186	1.0e-3

**Table 3:** Comparison of iteration time (s) on benchmark functions.

No.	Function name	PSO-NLCG time	NLCG time	CG time	PSO time
F1	Rosenbrock	0.0454	0.0426	0.0346	0.1452
F2	Quartic	0.0585	0.0444	0.0339	0.1580
F3	Sphere	0.0487	0.0461	0.0378	0.1153
F4	Rastrigin	0.0495	0.0443	0.0356	0.1718
F5	Griewank	0.0486	0.0412	0.0360	0.2345
F6	Ackley	0.0505	0.0422	0.0361	0.0769
F7	Alpine	0.0442	0.0462	0.0350	0.1632
F8	Schwefel	0.0476	0.0538	0.0340	0.1224
F9	Kowalik’s	0.0474	0.0426	0.0394	0.1305

As shown in Table 2; Fig. 2, for the unimodal functions F1 to F3, the local optimization algorithms (NLCG and CG) exhibit limited global optimization capability. Although the NLCG algorithm achieves a moderate improvement over the CG algorithm, its performance remains suboptimal. In contrast, both the PSO algorithm and the hybrid PSO-NLCG algorithm demonstrate excellent optimization accuracy, with the PSO-NLCG algorithm achieving results closer to the theoretical optima and exhibiting faster convergence.

For the multimodal functions F4 to F9, the global optimization performance of the PSO and PSO-NLCG algorithms significantly surpasses that of the local optimization methods (CG and NLCG). Among the four algorithms, the PSO-NLCG hybrid algorithm consistently produces solutions closest to the theoretical optima, confirming its superior optimization performance.

As shown in Table 3; Fig. 3, the PSO algorithm requires the longest computation time for both unimodal and multimodal benchmark functions, owing to its extensive global search process. The PSO-NLCG algorithm exhibits moderate runtime performance, while the local optimization methods (NLCG and CG) execute considerably faster due to their high efficiency in local refinement. Notably, the runtime difference between PSO-NLCG and the local methods remains relatively small. Thus, the PSO-NLCG algorithm achieves a favorable balance between optimization accuracy and computational efficiency, demonstrating robust overall performance.

### Experimental validation

**Fig. 4:**
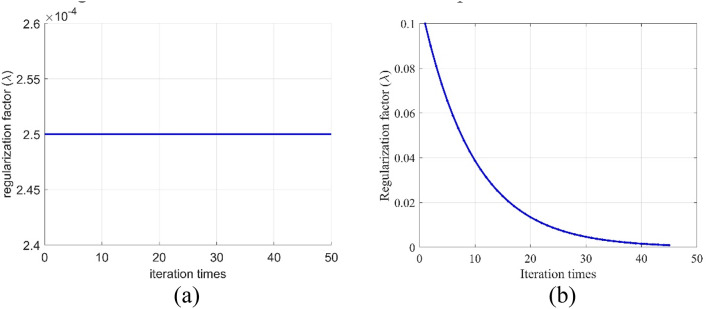
Comparison of Regularization Factor Iteration Curves.Subfigures (**a**) and (**b**) show the iteration curves for fixed regularization and adaptive regularization, respectively.

To evaluate the 3D visualization inversion performance of the proposed PSO-NLCG optimization combined with adaptive regularization, two Comparison method and one Proposed method were established. Comparison method 1 employed the conventional NLCG inversion algorithm, while Comparison method 2 utilized a fixed regularization inversion approach, in which the fixed regularization factor was set to the value obtained from the final iteration of the adaptive regularization process (The fixed regularization factor in Fig. 4a is $2.5 \times {10}^{-4}$). The Proposed method (PSO-NLCG) adopted the inversion algorithm combining PSO-NLCG optimization with adaptive regularization. The algorithm was configured with a population size of 200 and a total of 45 iterations, including two PSO-based presearch iterations. The initial regularization factor was set to 0.01 and decayed at a rate of 0.92, with a convergence threshold of ${10}^{-6}$. For a fair comparison, all three methods adopted the same initial model and parameter bounds. Specifically, the initial model was a homogeneous half-space whose conductivity was consistent with the background medium. The resistivity bounds were set to [10,10,10] and [80,80,80], and the thickness bounds were set to [20,20] and [100,130]. For Comparison method 1 (NLCG), the algorithm was run with a population size of 200 and a maximum of 45 iterations. The associated PSO-related parameters were set as ${w}_{\mathrm{m}\mathrm{a}\mathrm{x}}=0.9$, ${w}_{\mathrm{m}\mathrm{i}\mathrm{n}}=0.4$, and ${c}_{1}=1.49445$, ${c}_{2}=1.49445$. For Comparison method 2 (Fixed regularization), the population size was also set to 200 with a total of 45 iterations, and the line-search parameters were configured as an initial step length of $\alpha=1$, a reduction factor of $\rho=0.5$, and preset parameters ${c}_{1}={10}^{-4}$ and ${c}_{2}=0.9$.

In the TEM 3D visualization inversion experiments, a step-off waveform on the falling edge was adopted as the transmitting signal, with a transmitting current of 15 A, a turn-off time of 1 µs, and an observation time window ranging from 1 µs to 10 ms, in order to fully capture the electromagnetic field decay process.

Furthermore, Gaussian white noise with an amplitude of 2% was superimposed on the forward modeling theoretical data to simulate realistic exploration conditions.

#### Experiment 1

**Fig. 5:**
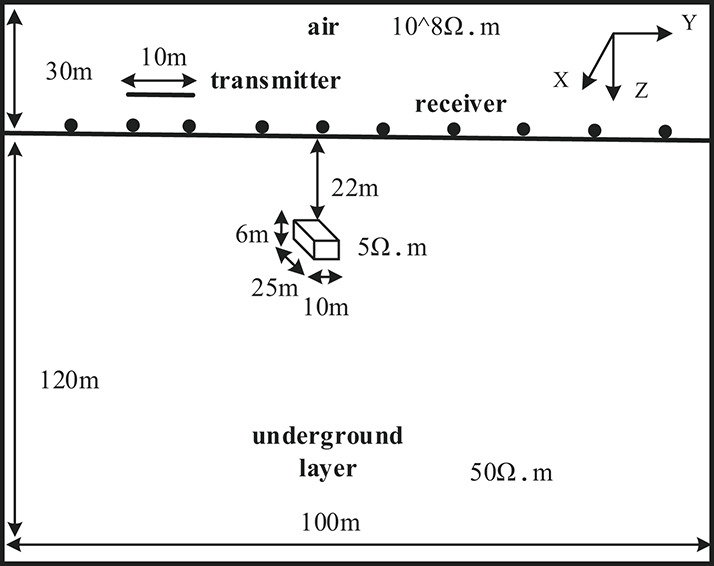
Schematic Diagram of Geoelectrical Model.

A geoelectrical model was constructed as shown in Fig. 5, with dimensions of 100 m × 100 m × 120 m and a background conductivity of 0.02 S/m. A horizontally oriented plate-shaped anomalous body (10 m × 25 m × 6 m) was embedded within the model, with its top buried at a depth of 22 m and its center located at coordinates (60, 46, − 25) m. The conductivity of the anomalous body was set to 0.2 S/m.

The observation systemre employed an overlapping loop configuration with a radius of 5 m and 50 turns. Ten survey lines (L1–L10) were arranged parallel to the x-axis, with both point and line spacing set to 10 m, forming a 10 × 10 grid with 100 measurement points. The initial model was defined as a homogeneous half-space with conductivity equal to that of the background.

**Fig. 6:**
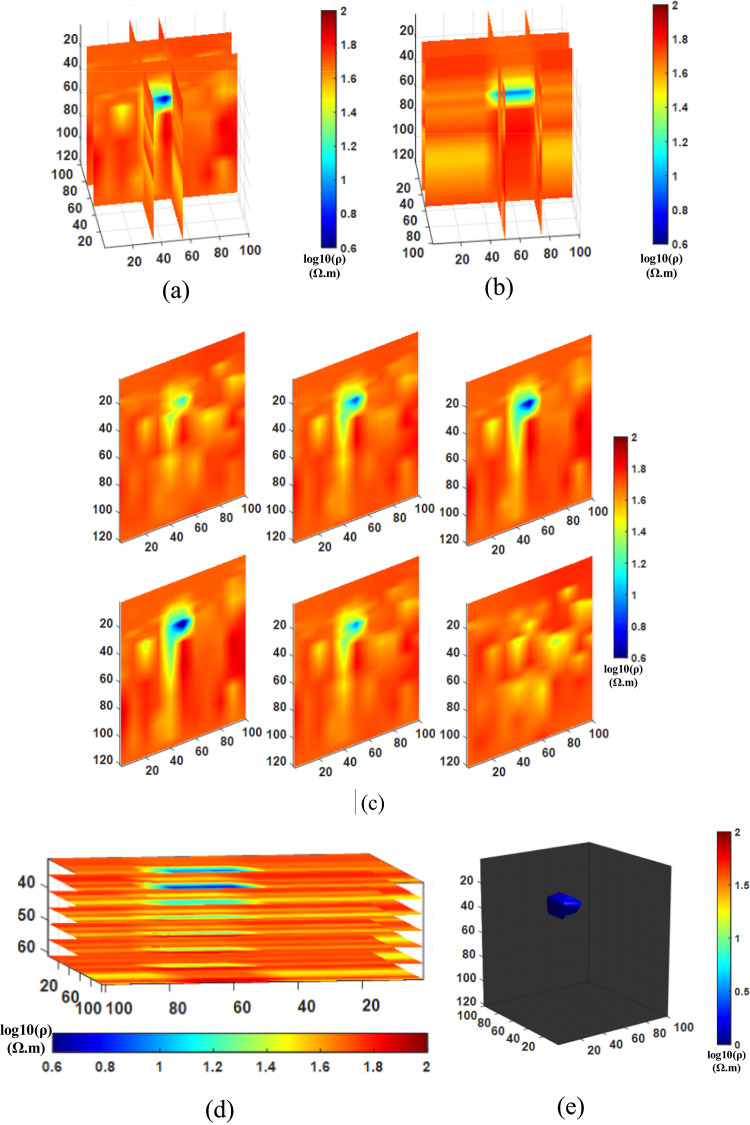
3D visualization Inversion Results for the NLCG Comparison method; (**a**) Front view of the grid- slice section (**b**) Side view of the gris-slice section (**c**) YOZ vertical slice view (**d)** XOY horizontal slice view (**e**) Isosurface 3D view.

**Fig. 7:**
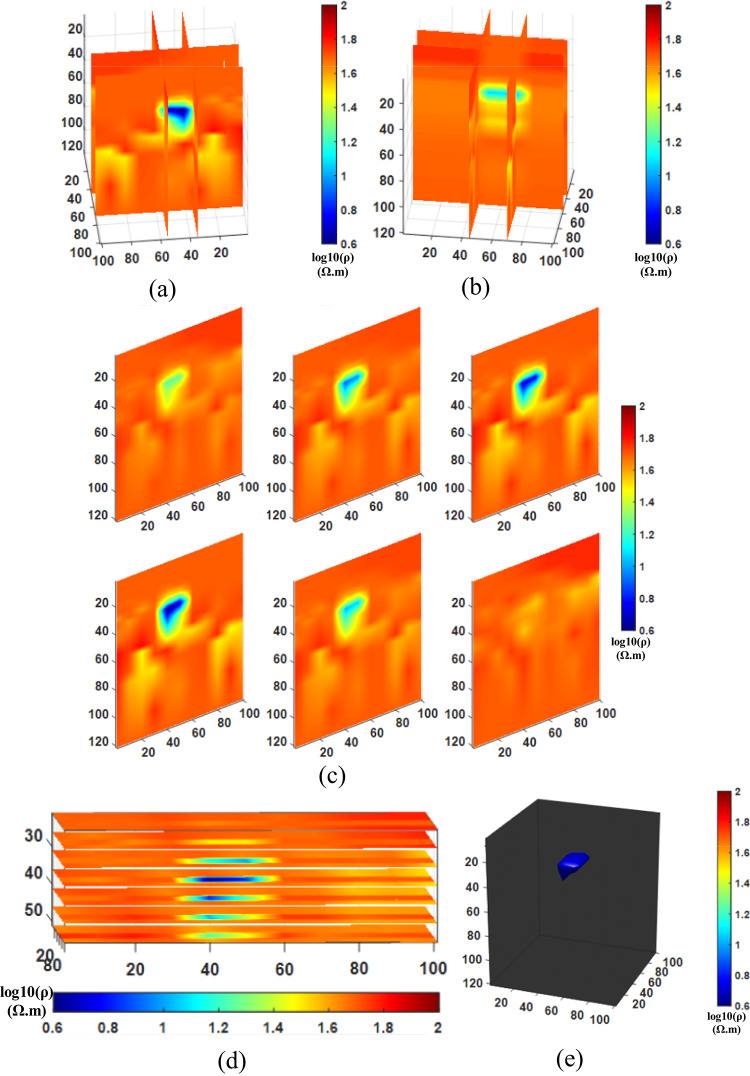
3D visualization Inversion Results for the Fixed-regularization Comparison method; (**a**) Front view of the grid-slice section (b) Side view of the grid- slice section (**c**) YOZ vertical slice view (**d**) XOY horizontal slice view (**e**) Isosurface 3D view.

**Fig. 8:**
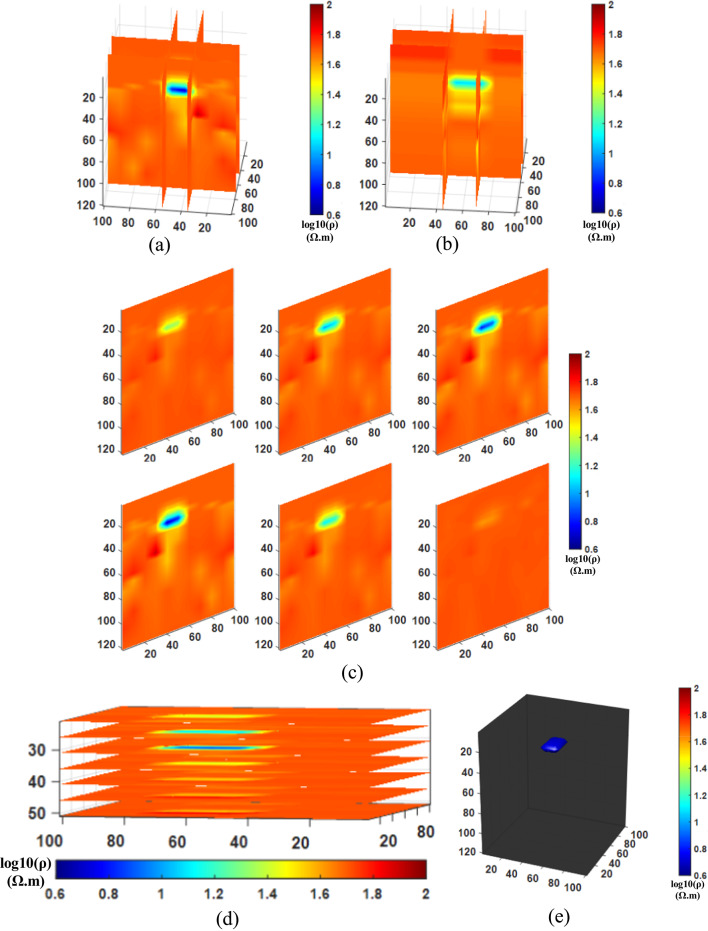
3D visualization Inversion Results for the PSO-NLCG adaptive regularization proposed method; (**a**) Front view of the grid-slice section (**b**) Side view of the grid-slice section (**c**) YOZ vertical slice view (**d**) XOY horizontal slice view (**e**) Isosurface 3D view.

After conducting the inversion experiments for Comparison method 1 (NLCG), Comparison method 2 (Fixed regularization), and the Proposed method (PSO-NLCG), the 3D visualization inversion results were obtained and are presented in Figs. 6 and 7, and 8. Each subfigure within these figures was constructed using different visualization approaches, as described below:

Figures (a) and (b) present the front and side views of the 3D grid-slice structure, generated by intersecting the YOZ planes at =35 m and x=55 m, and the XOZ planes at =50 m and y=75 m, respectively.

Subplot (c) shows vertical slice views along six typical YOZ planes within the range y = 30–100 m.

Subplot (d) displays horizontal slices of multiple typical XOY planes within the range y = 30–80 m.

Figure (e) illustrates the 3D envelope of the target body, constructed using isosurface rendering techniques.

The results in Fig. 6 indicate that the inversion results obtained by the NLCG local inversion algorithm exhibit significant distortion of the anomalous body. From the cross-shaped slices (see Fig. 6a and b), it can be observed that the background resistivity inversion is unstable, with abrupt variations occurring in regions where z > 40 m. The multi-position vertical slice (see Fig. 6c) further confirms this abrupt variation and clearly reveals that the geometric shape of the plate-like anomalous body is distorted, with its depth range significantly shifted downward. However, the horizontal slice (see Fig. 6d) and the isosurface 3D view (see Fig. 6e) illustrate the depth range of the anomalous body. In particular, Fig. e clearly shows that the volume of the plate-like anomalous body is approximately 20% larger than that of the standard model. Both the position and morphology of the anomaly exhibit varying degrees of deviation, indicating that the conventional gradient-based NLCG inversion algorithm is prone to being trapped in local minima, leading to excessive corrections of model parameters.

The results in Fig. 7 demonstrate that the inversion scheme with a fixed regularization factor results in blurred anomaly boundaries and slight deviations in its shape. From the grid-slice sections (see Fig. 7a and b) and the vertical slice (see Fig. 7c), it is clearly observed that the boundaries of the anomalous body are relatively blurred, and its depth extent is larger than that of the true model. The horizontal slices (see Fig. 7d) show the depth position of the anomaly, while the isosurface rendering (see Fig. 7e) further indicates an increased volume and minor deviations in the anomaly’s shape and spatial location.

In contrast, Fig. 8 illustrates the favorable inversion performance of the PSO-NLCG algorithm with adaptive regularization. The grid-slice sections (see Fig. 8a and b) and the vertical slice (see Fig. 8c) indicate that the resistivity distribution around the anomalous body is highly consistent with that of the background medium, and the depth extent of the anomalous body closely matches that of the standard model. The isosurface 3D view (see Fig. 8e) shows that the morphology and position of the anomalous body exhibit a high degree of spatial consistency with the standard model, further demonstrating the effectiveness of the PSO-NLCG adaptive regularization inversion algorithm in accurately recovering the electrical distribution of the target body.

**Fig. 9:**
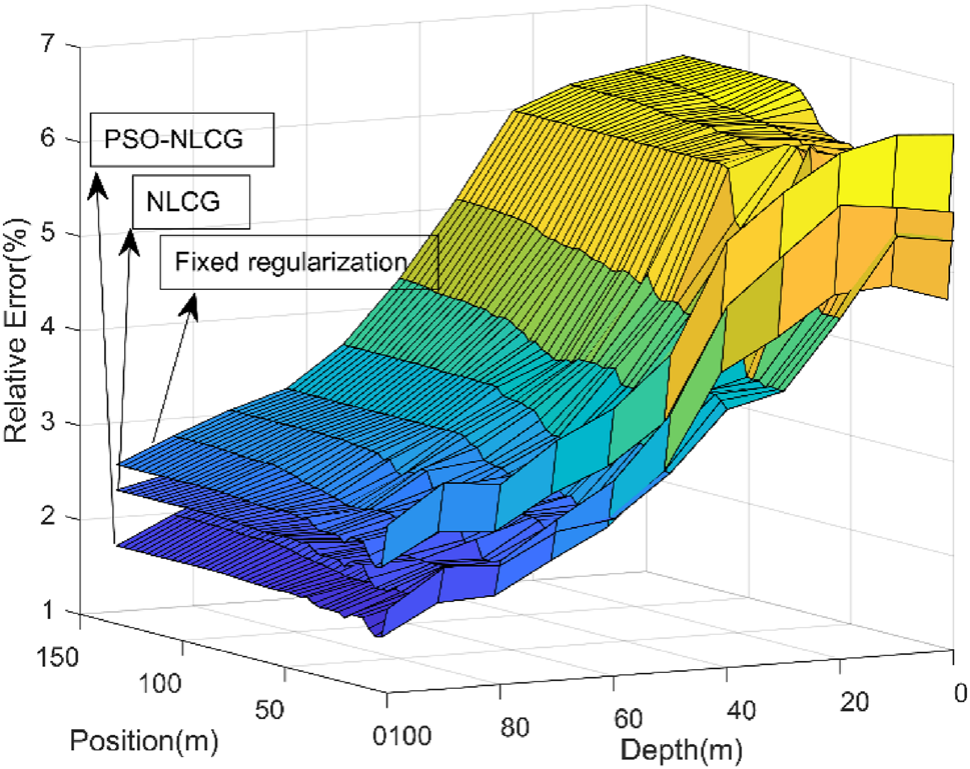
Inversion error maps for different inversion algorithms.

To more clearly quantify the inversion accuracy of different algorithms, an error analysis was performed for the fixed-regularization scheme, the NLCG algorithm, and the proposed PSO-NLCG algorithm. The analysis was conducted along the L5 survey line (directly above the anomalous body), where the relative error was defined as the discrepancy between the induced electromotive force (EMF) obtained at the final iteration of each inversion and the EMF derived from the forward response of the standard model. The relative error curves for the three methods are presented in Fig. 9. As shown in Fig. 9, several observations can be made. The inversion accuracy improves with increasing depth, and the relative errors of all three methods remain below 5%. For each algorithm, the inversion accuracy is nearly uniform among measurement points in the horizontal direction. Among the three schemes, PSO-NLCG yields the highest accuracy, whereas the fixed-regularization scheme produces the lowest. Overall, these results confirm that the PSO-NLCG inversion with adaptive regularization provides a clear advantage in terms of inversion accuracy.

#### Experiment 2

**Fig. 10:**
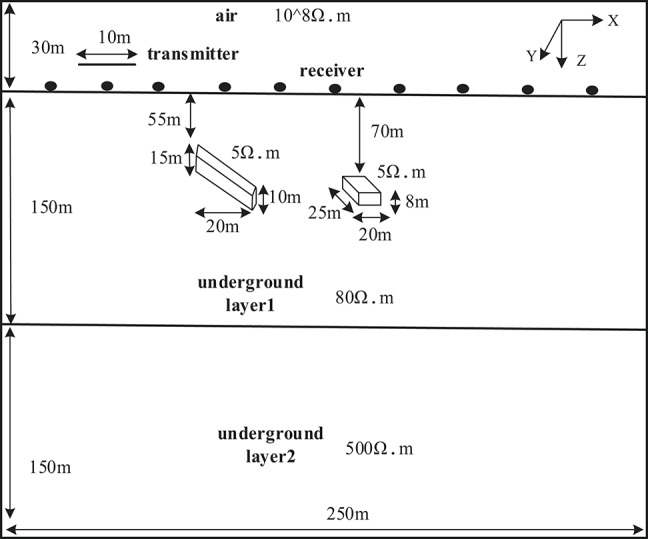
Schematic diagram of geoelectrical Model 2.

A multilayer geoelectrical model was constructed, as shown in Fig. 10, consisting of a shallow low-resistivity layer (250 m × 250 m × 150 m, 0.0125 S/m) overlying a deep high-resistivity layer of the same size (0.002 S/m). Two anomalous bodies with identical conductivity were embedded in the model: a horizontal tabular body measuring 20 m × 25 m × 8 m, with its top buried at 70 m and centered at (160, 112.5, − 74) m; and an inclined tabular body defined by four vertices in the X0Z plane at (75, 100, − 55) m, (75, 100, − 70) m, (95, 100, − 67) m, and (95, 100, − 77) m, with a width of 25 m and a top depth of 55 m. Both anomalies had a conductivity of 0.2 S/m. The observation system employed an overlapping loop configuration with an outer radius of 5 m and 50 turns. Ten survey lines (L1–L10) were arranged parallel to the y-axis, with both point and line spacing set to 25 m, forming a 10 × 10 grid of 100 measurement points. The initial model was defined as a layered structure with conductivity matching that of the background.

**Fig. 11:**
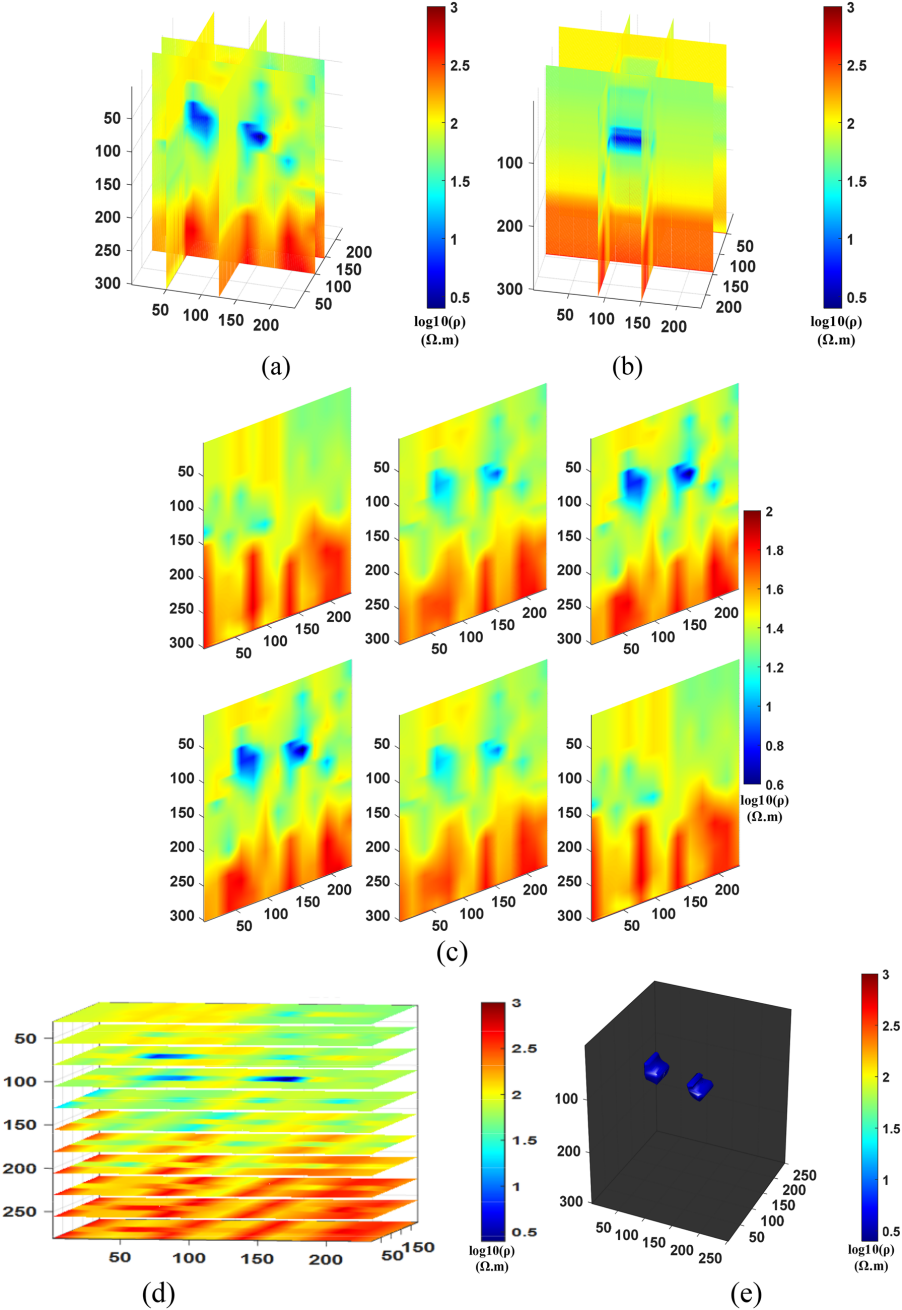
3D visualization Inversion Results for the NLCG Comparison method; (**a**) Front view of the grid- slice section (**b**) Side view of the gris-slice section (**c**) YOZ vertical slice view (**d**) XOY horizontal slice view (**e**) Isosurface 3D view.

**Fig. 12:**
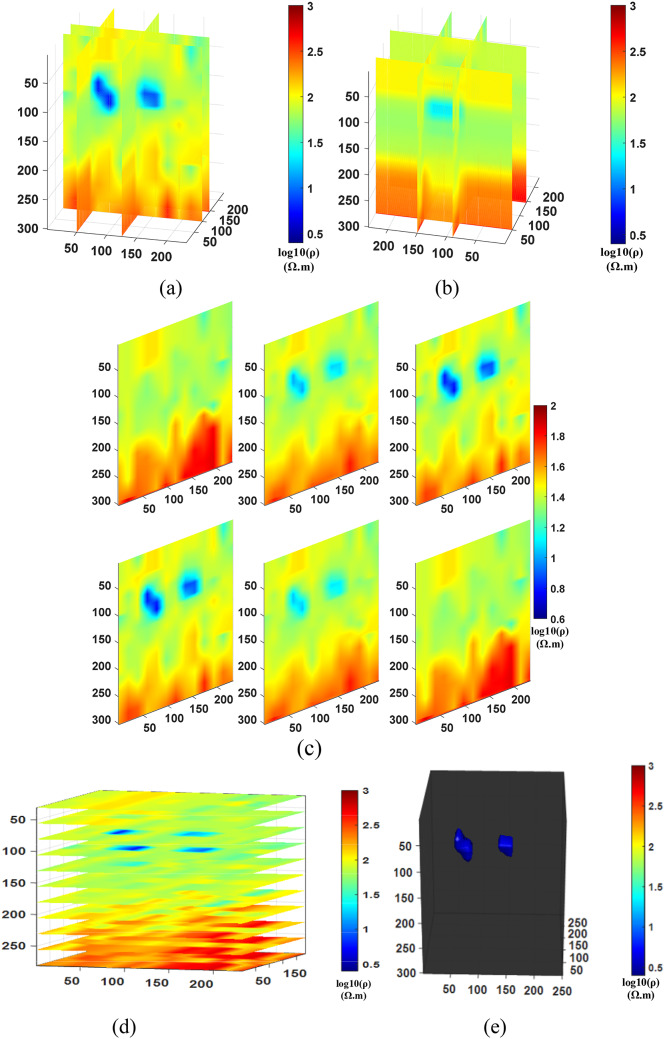
3D visualization Inversion Results for the Fixed-regularization Comparison method; (**a**) Front view of the grid-slice section (**b**) Side view of the grid- slice section (**c**) YOZ vertical slice view (**d**) XOY horizontal slice view (**e**) Isosurface 3D view.

**Fig. 13:**
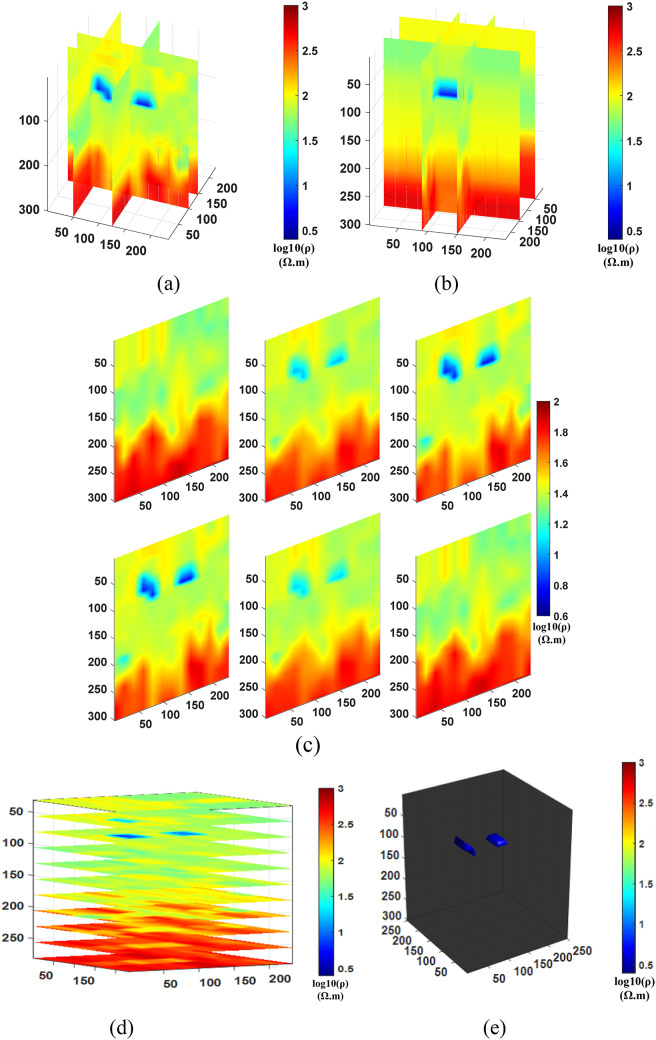
3D visualization Inversion Results for the PSO-NLCG adaptive regularization proposed method; (**a**) Front view of the grid-slice section (**b**) Side view of the grid-slice section (**c**) YOZ vertical slice view (**d**) XOY horizontal slice view (**e**) Isosurface 3D view.

Inversion experiments were conducted separately for Comparison method 1 (NLCG), Comparison method 2 (Fixed regularization), and the Proposed method (PSO-NLCG), the 3D visualization inversion results were obtained and are presented in Figs. 11 and 12, and 13. Each subfigure within these figures was constructed using different visualization approaches, as described below:

Subplots (a) and (b) show front and side views of cross-shaped slices, created by extracting YOZ planes at x = 50 m and x = 145 m, and XOZ planes at y = 90 m and y = 150 m, respectively.

Subplot (c) presents vertical slice views along six typical YOZ planes within the range y = 30–200 m.

Subplot (d) displays horizontal slices of multiple XOY planes within the range y = 30–300 m.

Subplot (e) shows a 3D envelope of the target bodies, constructed using isosurface rendering techniques.

As shown in Fig. 11, the inversion results obtained using the local NLCG algorithm exhibit significant morphological distortions and positional deviations of the anomalous bodies. The front cross-shaped slice (see Fig. 11a) and the vertical slice (see Fig. 11c) clearly show that the depth extents of both the inclined and horizontal anomalies are overestimated. Noticeable deviations are observed in both shape and position. Additionally, the inverted resistivity values in the deep background are underestimated, failing to reflect the high-resistivity characteristics of the deeper layer in the multilayer model.The horizontal slice (see Fig. 11d) and isosurface rendering (see Fig. 11e) reveal the overall spatial extent of the targets. Figure 11e further illustrates the detailed morphology and distribution of the anomalies. Both the inclined and horizontal anomalies appear enlarged by more than 10% compared to the reference model, and clear discrepancies are observed in their shapes and locations. These results further highlight the limitations of the gradient-based NLCG inversion algorithm in accurately reconstructing the geometry and position of multiple subsurface targets.

The results in Fig. 12 indicate that the inversion outcome using a fixed regularization factor is generally satisfactory. However, the cross-shaped slice view (see Fig. 12a) and the vertical slice view (see Fig. 12c) show that both the inclined and horizontal tabular anomalies exhibit expanded anomalous extents compared to the reference model. Additionally, the boundary between the shallow and deep background resistivity layers is not clearly resolved. The horizontal slice view (see Fig. 12d) and the isosurface rendering (see Fig. 12e) reveal the depth extent of the anomalous bodies (see Fig. 12e) indicates that the shape of the horizontal tabular anomaly is not accurately recovered through inversion, and the volume of the multi-target body is overestimated.

In contrast, Fig. 13 demonstrates that the inversion results obtained using the PSO-NLCG algorithm with adaptive regularization are significantly improved. The cross-shaped slice views (see Fig. 13a and b) and the vertical slice view (see Fig. 13c) indicate that the anomalous extent of the multi-target body is more consistent with the dimensions of the reference model. Additionally, the boundary between the shallow and deep background resistivity layers is more clearly defined. The horizontal slice view (see Fig. 13d) and the isosurface rendering (see Fig. 13e) show that the depth extent of the anomalies more closely matches the depth dimensions of the reference model. Moreover, Fig. 13e more clearly illustrates the shape and spatial distribution of the multi-target body, which closely aligns with that of the reference model. These results further demonstrate the effectiveness of the PSO-NLCG inversion algorithm with adaptive regularization in accurately recovering the spatial distribution of multi-target bodies.

**Fig. 14:**
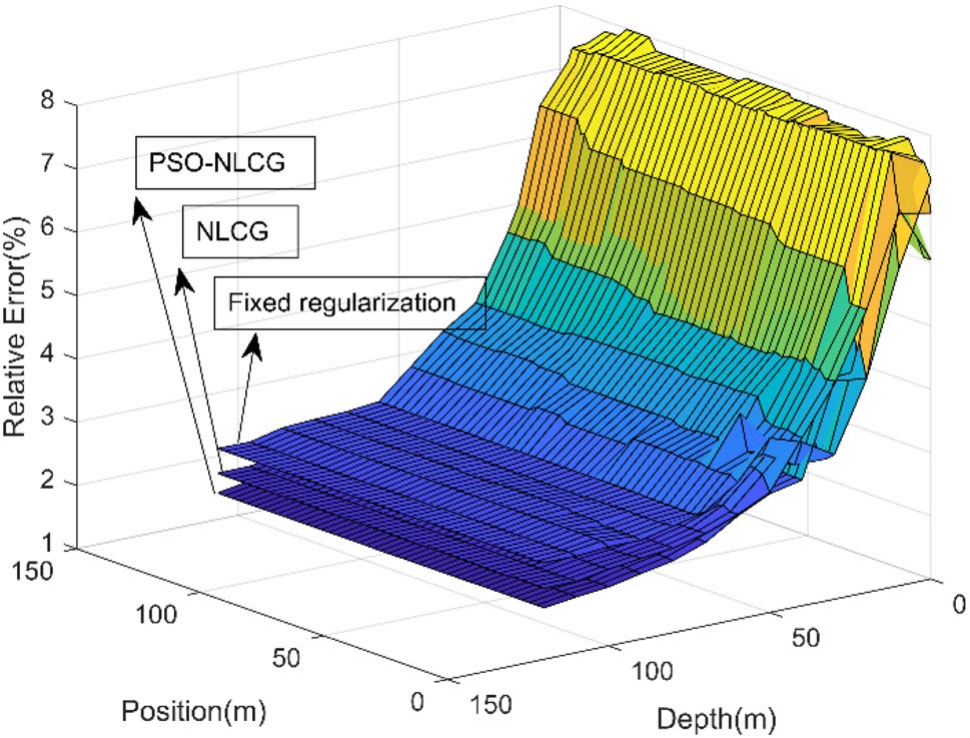
Inversion error maps for different inversion algorithms.

To further demonstrate the superiority of the inversion algorithm, an error analysis was conducted for the fixed-regularization scheme, the NLCG algorithm, and the proposed PSO-NLCG algorithm. The analysis was performed along the L4 survey line (located above the anomalous body), where the relative error was calculated by comparing the induced electromotive force (EMF) obtained at the final iteration of each inversion with the EMF from the forward response of the standard model. The relative error results for the three methods are presented in Fig. 14. Several conclusions can be drawn from Fig. 14. First, because a different survey line is selected from that in Experiment 1, the relative error curves exhibit slight differences compared with those in Fig. 9. Nevertheless, the remaining observations are essentially consistent with the conclusions of Experiment 1, which further confirms the stability of the inversion schemes and highlights the advantage of the PSO-NLCG inversion with adaptive regularization in terms of inversion accuracy.

### Field data application and validation

**Fig. 15:**
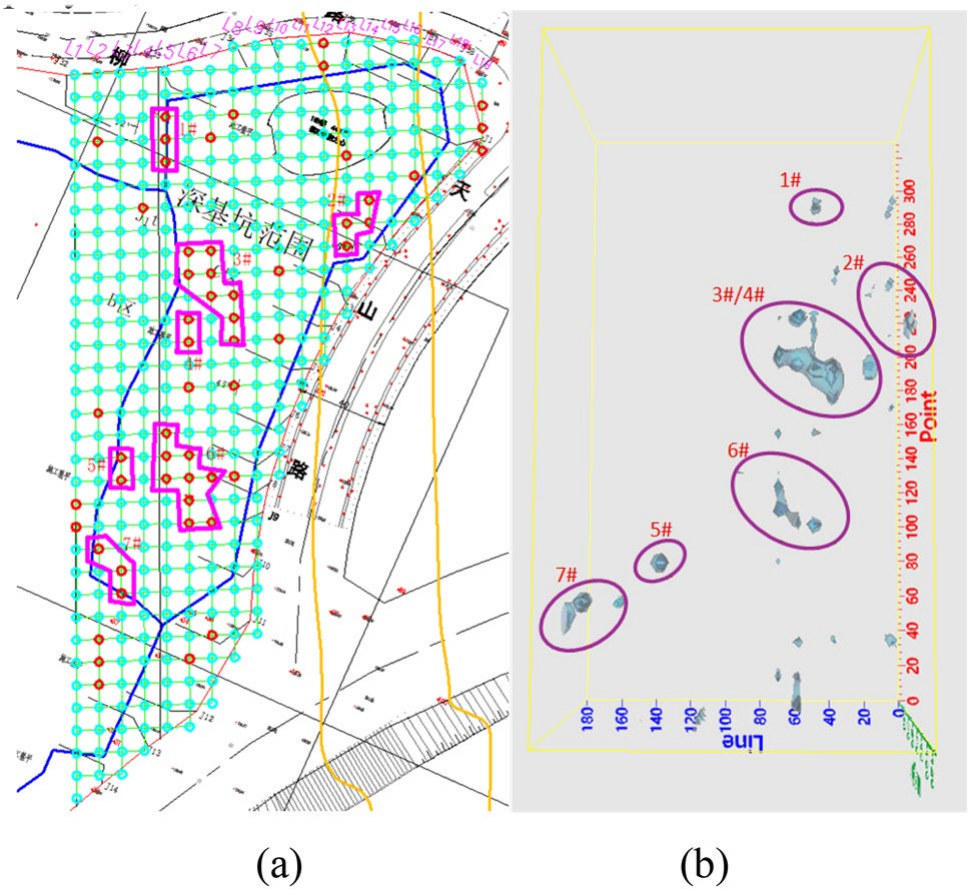
(**a**) Actual distribution of underground anomalies at a construction site, (**b**) Three-dimensional inversion results obtained using the proposed method.

To further validate the effectiveness of the proposed method, transient electromagnetic (TEM) data were acquired prior to construction at a building site with known anomalies, and the collected data were subsequently verified through 3D inversion. The known underground anomalies in the goaf area are illustrated in Fig. 15a. As shown in the figure, a total of seven underground anomalies have been identified and labeled from No. 1 to No. 7. A total of 19 survey lines were conducted, with a grid spacing of 10 m × 10 m. The TEM data were collected using a DKTEM-18 system. During signal acquisition, the transmitter current was set to 50 A with a pulse width of 10 ms. A square transmitting–receiving coil with dimensions of 2 m × 2 m was employed, and each measurement was stacked 64 times.

**Fig. 16:**
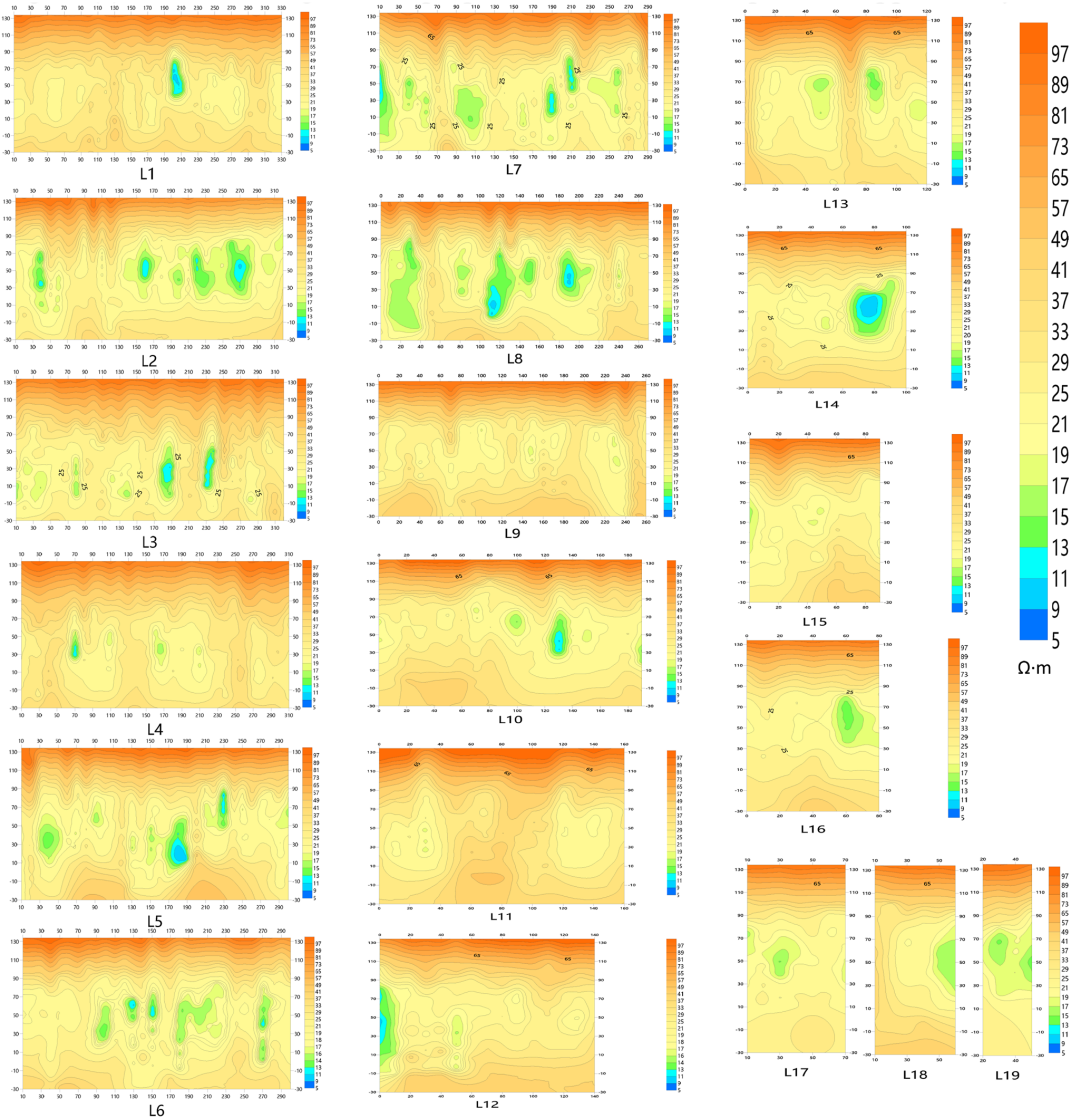
Profile charts from L1 to L19.

**Fig. 17:**
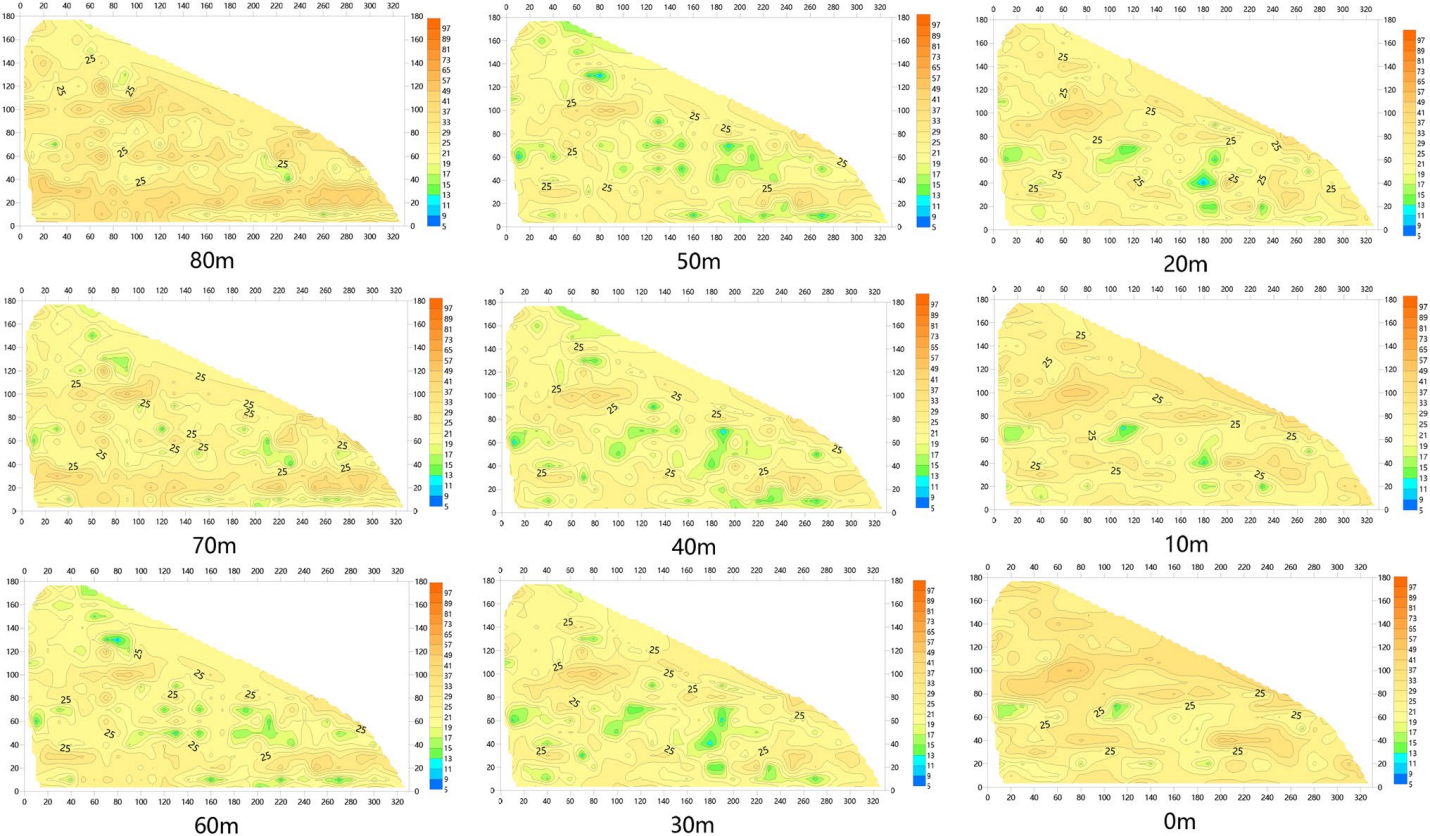
The slice resistivity profiles with different depth.

After inversion of the acquired data using the method proposed in this paper, nineteen profile charts named L1-L19 and nine slice resistivity profiles with different depth were made in Surfer, which can be seen from Figs. 16 and 17 respectively. The figures demonstrate that, the anomaly locations of the sectional view from L1 to L19 are almost the same as the underground anomalies shown in Fig. 15a, and there is no anomaly in section L9 and L11. what’s more, from the slice resistivity profile with different depth can be seen that, the depth of the underground anomalies is mainly located from underground 20 m to 60 m.

Based on the slice maps mentioned above, three-dimensional visualizations were generated using Voxler software, and the corresponding top-view representation is shown in Fig. 15b. As can be observed from the figure, due to the close spatial proximity between anomalies No. 3 and No. 4 in Fig. 15a, these two anomalies cannot be clearly distinguished in the inverted three-dimensional results. Nevertheless, the remaining anomaly regions are effectively identified and show good spatial correspondence with the anomaly locations in Fig. 15a, demonstrating that the proposed method exhibits practical applicability.

## Conclusions

This work proposes a 3D TEM inversion method based on PSO-NLCG joint optimization method and adaptive regularization method, and constructs a complete framework from theory to 3D visualization inversion. In terms of algorithm innovation, by integrating the global search capability of PSO with the local fine adjustment capability of NLCG gradient optimization, the stability and accuracy of inversion have been effectively improved, and the risk of falling into local optima has been reduced. Compared with traditional NLCG and fixed regularization methods, the PSO-NLCG algorithm performs better in restoring the morphology, position, and spatial distribution of anomalous bodies, with clear boundaries, accurate positioning, and smaller overall inversion errors. Therefore, this method has good application prospects in improving the quality and reliability of 3D TEM inversion.

## Supplementary Information

Below is the link to the electronic supplementary material.

Supplementary Material 1.

Supplementary Material 2.

## Data Availability

All data generated or analysed during this study are available from the corresponding author upon reasonable request.
