# Supplementary material for: A 3D inversion method of TEM combining PSO-NLCG optimization and adaptive regularization
Source: Sci Rep. 2026 Apr 9;16:16115. doi: 10.1038/s41598-026-48117-x (PMC13199583; doi:10.1038/s41598-026-48117-x)
Supplement: Supplementary file 1 — Supplementary Material 1. [file 41598_2026_48117_MOESM1_ESM.docx]

Figure 6：

Figure 7：

Figure 8：

Figure 11：

Figure 12：

Figure 13：
